# Proteomics of Breast Muscle Tissue Associated with the Phenotypic Expression of Feed Efficiency within a Pedigree Male Broiler Line: I. Highlight on Mitochondria

**DOI:** 10.1371/journal.pone.0155679

**Published:** 2016-05-31

**Authors:** Byung-Whi Kong, Kentu Lassiter, Alissa Piekarski-Welsher, Sami Dridi, Antonio Reverter-Gomez, Nicholas James Hudson, Walter Gay Bottje

**Affiliations:** 1 Department of Poultry Science, Center of Excellence for Poultry Science, University of Arkansas, Fayetteville AR 72701, United States of America; 2 CSIRO Livestock Industries, Queensland Bioscience Precinct, 306 Carmody Road, St. Lucia, QLD 4067, Australia; University of California Davis, UNITED STATES

## Abstract

As feed represents 60 to 70% of the cost of raising an animal to market weight, feed efficiency (the amount of dry weight intake to amount of wet weight gain) remains an important genetic trait in animal agriculture. To gain greater understanding of cellular mechanisms of feed efficiency (FE), shotgun proteomics was conducted using in-gel trypsin digestion and tandem mass spectrometry on breast muscle samples obtained from pedigree male (PedM) broilers exhibiting high feed efficiency (FE) or low FE phenotypes (n = 4 per group). The high FE group had greater body weight gain (P = 0.004) but consumed the same amount of feed (P = 0.30) from 6 to 7 wk resulting in higher FE (P < 0.001). Over 1800 proteins were identified, of which 152 were different (*P* < 0.05) by at least 1.3 fold and ≤ 15 fold between the high and low FE phenotypes. Data were analyzed for a modified differential expression (DE) metric (Phenotypic Impact Factors or PIF) and interpretation of protein expression data facilitated using the Ingenuity Pathway Analysis (IPA) program. In the entire data set, 228 mitochondrial proteins were identified whose collective expression indicates a higher mitochondrial expression in the high FE phenotype (binomial probability *P* < 0.00001). Within the top up and down 5% PIF molecules in the dataset, there were 15 mitoproteome proteins up-regulated and only 5 down-regulated in the high FE phenotype. Pathway enrichment analysis also identified mitochondrial dysfunction and oxidative phosphorylation as the number 1 and 5 differentially expressed canonical pathways (up-regulated in high FE) in the proteomic dataset. Upstream analysis (based on DE of downstream molecules) predicted that insulin receptor, insulin like growth receptor 1, nuclear factor, erythroid 2-like 2, AMP activated protein kinase (α subunit), progesterone and triiodothyronine would be activated in the high FE phenotype whereas rapamycin independent companion of target of rapamycin, mitogen activated protein kinase 4, and serum response factor would be inhibited in the high FE phenotype. The results provide additional insight into the fundamental molecular landscape of feed efficiency in breast muscle of broilers as well as further support for a role of mitochondria in the phenotypic expression of FE.

Funding provided by USDA-NIFA (#2013–01953), Arkansas Biosciences Institute (Little Rock, AR), McMaster Fellowship (AUS to WB) and the Agricultural Experiment Station (Univ. of Arkansas, Fayetteville).

## Introduction

The projected doubling of the human population by 2060 will require a 100% increase in food production coming from plants and animals with most of this increase coming from new technology and greater efficiency [1]. In animal production agriculture, feed is the highest input cost (50 to 70% of total) in raising an animal to market weight and feed costs can spike as observed during the severe drought in the US in 2012 [2]. Great strides have been made in animal agriculture production efficiency through the selection of animals for feed efficiency (FE, weight gained to feed consumed), feed conversion ratio (FCR, feed to gain), or residual feed intake (RFI, the actual amount of feed intake that is above or below predicated feed intake in a group of animals). These are all labor intensive but effective methods that require measuring feed intake and body weight gain on individual animals. Development of biomarker selection tools to be used in commercial breeding programs can contribute significantly towards increasing animal production efficiency.

Global gene expression studies have been conducted to gain a greater understanding of the cellular basis of FE in three different broiler models. Using individually phenotyped birds, gene expression has been investigated in; 1) duodenal tissue in broilers selected for low and high RFI [3], 2) breast muscle tissue in commercial broilers [4], and 3) breast muscle within a pedigree male (PedM) broiler line [5]. Based on the cDNA microarray results [5, 6], we hypothesized that gene expression in the low FE phenotype resulted from inherent gene expression that was modulated by mitochondrial ROS [7]. Higher oxidative stress was observed in the low FE PedM broiler phenotype in the form of higher mitochondrial reactive oxygen species (ROS) production and pervasive protein oxidation in isolated mitochondrial and whole tissue homogenates [8–10]. In contrast, Zhou et al. [4] hypothesized that increased oxidative stress in high FE commercial broilers was due to higher inflammatory gene expression (cytokines or myokines) associated with muscle remodeling and development.

Despite the potential of transcriptome analyses for discovering important genes and pathways associated with economically important production traits, gene transcript profiles provide only part of the picture as they may be subject to feedback control by their protein products. Potential drawbacks of transcriptome analyses include inconsistencies of corresponding protein expression levels [11–15] and lack of information on post-translational modifications (e.g. phosphorylation). Proteomic approaches are promising as they can circumvent some of the issues associated with transcriptomics. Various proteomic studies in poultry have recently emerged including reports on muscle [16, 17], eggs [18–20], and liver [21] as well as host-virus interactions [22–26]. Muscle proteomics in livestock has been extensively studied for physiological relevance associated with specific diseases, metabolic conditions, and production traits (See review by Picard et al. [27]). Most studies have relied on two dimensional gel electrophoresis (2DGE)-based protein identification methods. The 2DGE of crude tissue extracts usually underestimates the presence of low abundance elements, integral membrane proteins, regulatory proteins (e.g. transcription factors), and components with very high molecular masses in complex tissues, such as muscle [28] that contain very high levels of muscle structural subunits.

Shotgun proteomics based on high-resolution mass spectrometry (MS) allows quantification of thousands of proteins along with their modifications, localization, turnover, and interaction partners [29, 30]. Shotgun proteomics does not focus on specific proteins and thus offers a hypothesis-free and systems-wide analysis that complements antibody-based approaches. Advances in all areas of the MS-based proteomics workflow, including sample preparation, liquid chromatography (LC)-MS and computational analysis, have made it a preferred analytic tool to characterize protein dynamics in multiple functional dimensions; and proteomics based on LC-MS has essentially replaced previous tools such as 2DGE. The shotgun proteomics method has been used in poultry [22].

The major goal of this study was to conduct experiments in order to gain new insight into the cellular basis of FE by analysis of proteomic data obtained in PedM broilers exhibiting high or low FE phenotypes. We screened the data for patterns of differential expression (DE) and assessed the mitochondrial proteome in particular detail. We used DE and cluster analysis to identify groups of proteins expressed at different levels between the two groups of birds. Furthermore, a commercial software program (Ingenuity Pathway Analysis, IPA; Qiagen, Valencia, CA) was used to develop predictions of activation or inhibition of upstream regulators, molecules, and pathways of DE proteins. The analysis was conducted on the same set of samples that were used for microarray examination of global gene expression associated with FE [5], thus starting to develop a proteogenomic profile associated within this particular FE model.

## Materials and Methods

### Ethics Statement

The present study was conducted in accordance with the recommendations in the guide for the care and use of laboratory animals of the National Institutes of Health. All procedures for animal care complied with the University of Arkansas Institutional Animal Care and Use Committee (IACUC): Protocol #14012.

### Animals and tissue (breast muscle) collection

Proteomic analysis was conducted on a subset of breast muscle samples previously used in global gene expression analysis [5]. Phenotyping of animals in this study followed procedures outlined previously [8]. Briefly, feed efficiency (FE, amount of body weight gain/amount of feed consumed) was determined between 6 and 7 wk of age on a group of 100 pedigree male (PedM) broilers housed in individual cages. Birds were provided access to feed and water ad libitum. All birds received the same corn-soybean based diet (20.5% protein, 3,280 kcal/kg) during the feed efficiency trial. From this group of 100 birds with the highest and lowest FE were selected. Feed efficiencies for the low- and high-FE groups (n = 4 per group) in the present study were 0.46 ± 0.01 and 0.65 ± 0.01, respectively. Higher efficiency was obtained by greater weight gain without a difference in feed intake during the week of FE phenotyping. The difference in mean FE (0.19) is identical to that reported in our initial investigation [8]. Birds were humanely killed, tissues were quickly removed, and flash frozen in liquid nitrogen, and stored at -80°C.

### Protein extraction

**Fig 1:**
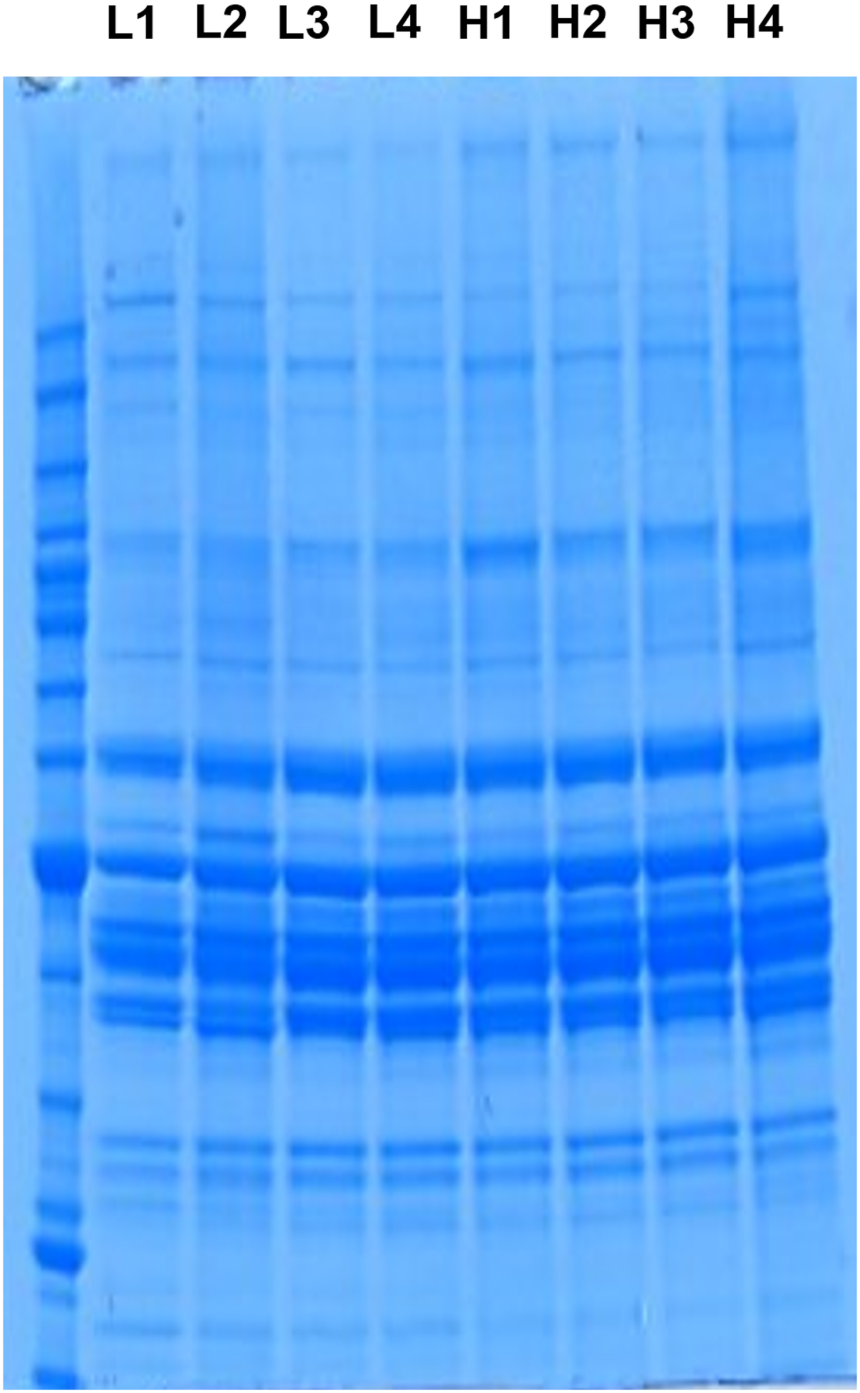
SDS-PAGE gel electrophoresis in broiler breast muscle. Protein bands prior to protein extractions of breast muscle obtained from pedigree broiler breeder males exhibiting low feed efficiency (L) or high feed efficiency (H) (n = 4 per group). From this gel, a total of 25 slices were obtained and subjected to tryptic digestion prior to conducting shotgun proteomics.

Protein extraction methodology followed procedures by Iqbal et al. [31] with modifications. Briefly, total proteins were extracted from breast muscles of 4 birds per group (high and low FE), which were used in cDNA microarray [5]. Approximately 100 mg of frozen muscle tissue was minced and homogenized in 700 μl of ice-cold tissue lysis buffer containing 150 mM NaCl, 0.1% Triton X-100, 0.5% sodium deoxycholate, 0.1% SDS, 50 mM Tris-HCl pH 8.0, and protease and phosphatase inhibitors (Life Technologies, Grand Island, NY) with 0.9–2.0 mm stainless steel bullet blender beads (Next Advance, Averill Park, NY). The samples were centrifuged at 4°C for 20 min at 14,000xg and the supernatant was carefully collected, aliquoted and stored at -80°C until use. Protein concentration was determined by Bradford assay using a Synergy HT multi-mode microplate reader (BioTek, Winooski, VT). Extracted proteins were analyzed by SDS-PAGE gel electrophoresis (Life Technologies, Grand Island, NY) to assess protein quality by the presence of distinct protein bands (Fig 1).

### Shotgun proteomics

Extracted individual proteins were subjected to shotgun proteomics analysis by in-gel trypsin digestion and tandem mass spectrometry (MS/MS) at the University of Arkansas Medical Science (UAMS) Proteomics Core Lab (Little Rock, AR). In total, 25 gel slices per sample were analyzed. Raw mass spectrometric data were analyzed by database searching using the Mascot (Matrix Science, Boston, MA) search engine and the UniProtKB (http://www.uniprot.org/help/uniprotkb) database. Search results were compiled using Scaffold program (Proteome Software, Portland, OR).

### Statistics and Bioinformatic Analyses

**Fig 2:**
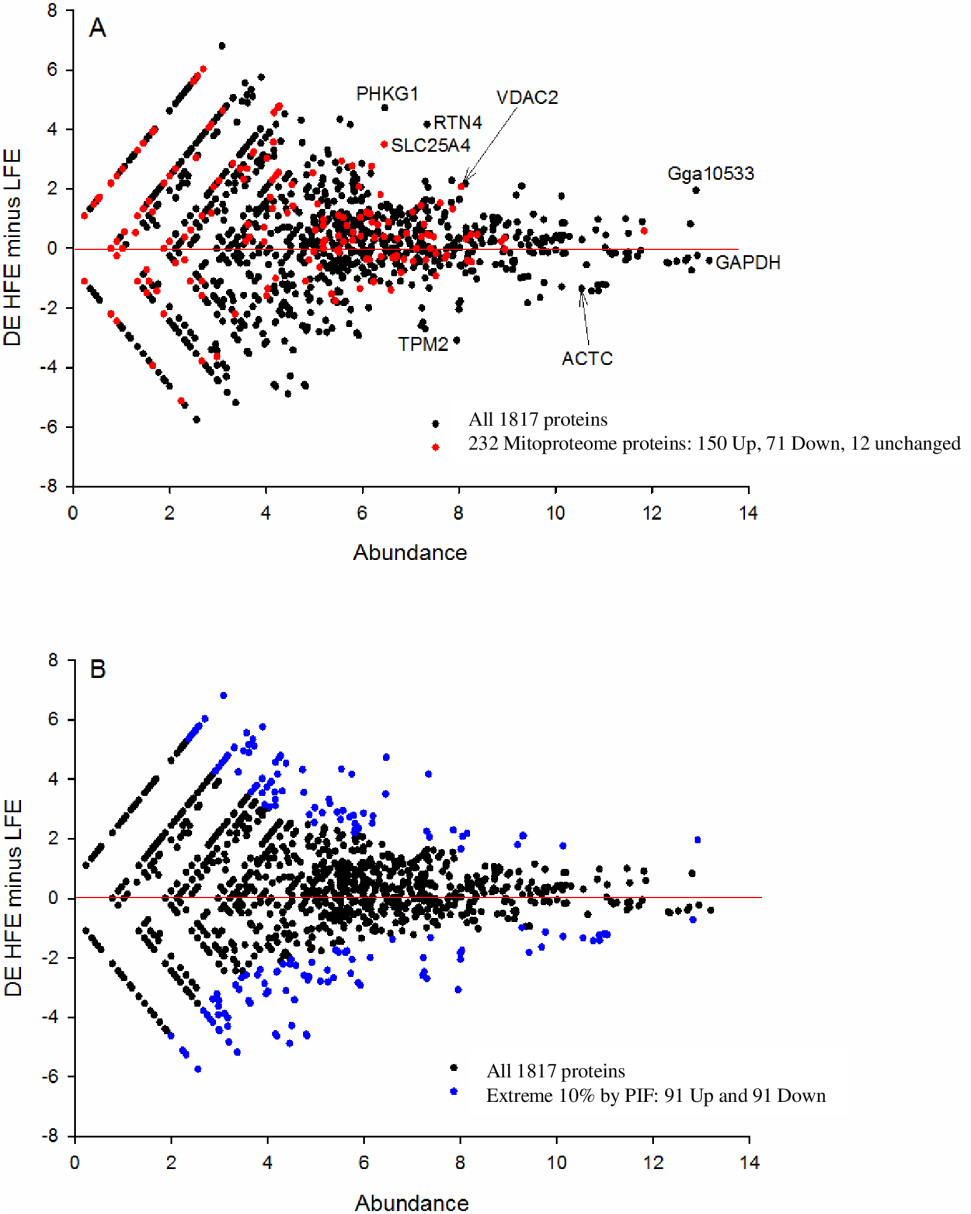
The protein abundance for the difference (Minus [M] for high FE minus low FE) and protein abundance alone (A) for all proteins. The MA plot is consistent with a reduction in slow fiber subunits (TPM2, ACTC) in the high FE (HFE) and an increase in mitochondrial regulators of muscle energy supply in the form of ATP (SLC25A4) and creatine phosphate (VDAC2). GAPDH is the most abundant protein we identified. Further, the mitoproteome (highlighted in red, Fig 2A) is skewed towards the HFE consistent with a higher mitochondrial content. Highly differentially expressed proteins were identified by a metric called Phenotypic Impact Factor (PIF), with the extreme 10% highlighted in blue (Fig 2B). Abbreviations: (RTN4), reticulon 4; (PHKG1), phosphorylase kinase gamma 1; (SLC26A4), adenine nucleotide translocase 1; (ACTC), alpha actin cardiac; (Gga), golgi associated gamma adaptin; (GAPDH) glyceraldyhde 3-phosphate dehydrogenase; (TPM2), tropomyosin 2.

Data were normalized based on total spectral counts for each individual sample. Evidence of success in normalization of samples is provided in S1 Fig showing no differences in actin, myosin or tubulin expression that serve as standard internal house-keeping proteins. Expression of GAPDH, often used as an internal reference check, was down-regulated (-1.45 fold, P < 0.05) in the high compared to low FE group (See Table 1 below). To generate MA plots, we added a nominal 0.05 to all protein abundance values in order to handle the presence of 0’s, log_2_ transformed the data to stabilize the variance, and computed average (A) and minus (M) at the group level (high FE minus low FE) for all 1817 proteins (See Fig 2A). The A values were 0 justified by adding 4 to all values.

**Table 1:** Protein expression (Fold Change) in breast muscle obtained from a single male broiler breeder line individually phenotyped for high or low FE (n = 4 per group). Positive and negative fold change values indicates proteins that were up-regulated in high FE and low FE broiler males, respectively, with significance level designated by *P*-values.

Symbol	Entrez Gene Name	Fold Change	P value
***Up-regulated proteins (104 total) in breast muscle obtained from Pedigree Broiler Males exhibiting high FE*.**			
KRT15	keratin 15	13.70	0.036
CACNA1S	calcium channel, voltage-dependent, L type, alpha 1S subunit	12.08	0.004
IDE	insulin-degrading enzyme	11.69	0.004
SLC25A4	solute carrier family 25 (mitochondrial carrier; adenine nucleotide translocator), member 4	10.32	0.002
PHKG1	phosphorylase kinase, gamma 1 (muscle)	9.81	0.005
CAV1	caveolin 1, caveolae protein, 22kDa	9.40	0.009
FABP4	fatty acid binding protein 4, adipocyte	9.31	0.012
CKB	creatine kinase, brain	8.91	0.003
PPA2	pyrophosphatase (inorganic) 2	8.85	0.007
ARF4	ADP-ribosylation factor 4	8.81	0.004
NDUFV2	NADH dehydrogenase (ubiquinone) flavoprotein 2, 24kDa	8.77	0.003
PTRH2	peptidyl-tRNA hydrolase 2	7.78	0.001
EIF4G2	eukaryotic translation initiation factor 4 gamma, 2	7.68	0.015
SNAP23	synaptosomal-associated protein, 23kDa	7.55	0.008
APRT	adenine phosphoribosyltransferase	7.28	0.038
SAR1B	secretion associated, Ras related GTPase 1B	6.99	0.019
OTUB1	OTU deubiquitinase, ubiquitin aldehyde binding 1	6.66	0.051
G3BP1	GTPase activating protein (SH3 domain) binding protein 1	6.59	0.002
MYO18A	myosin XVIIIA	6.50	0.017
MTHFD1L	methylenetetrahydrofolate dehydrogenase (NADP+ dependent) 1-like	6.24	0.047
RAP1B	RAP1B, member of RAS oncogene family	6.02	0.001
IPO7	importin 7	5.94	0.006
ARF1	ADP-ribosylation factor 1	5.75	0.010
GLRX3	glutaredoxin 3	5.66	0.004
NDUFB8	NADH dehydrogenase (ubiquinone) 1 beta subcomplex, 8, 19kDa	5.64	0.005
PHKB	phosphorylase kinase, beta	5.49	0.033
SNX3	sorting nexin 3	5.38	0.034
DHRS7C	dehydrogenase/reductase (SDR family) member 7C	5.13	0.058
NDUFS7	NADH dehydrogenase (ubiquinone) Fe-S protein 7, 20kDa (NADH-coenzyme Q Red.)	4.79	0.049
SKP1	S-phase kinase-associated protein 1	4.78	0.054
PRPS1	phosphoribosyl pyrophosphate synthetase 1	4.60	0.027
TARS	threonyl-tRNA synthetase	4.52	0.017
UBE2H	ubiquitin-conjugating enzyme E2H	4.29	0.028
PTRF	polymerase I and transcript release factor	4.25	0.006
UQCRC1	ubiquinol-cytochrome c reductase core protein I	4.11	0.059
NME3	NME/NM23 nucleoside diphosphate kinase 3	4.06	0.019
GNB2L1	guanine nucleotide binding protein (G protein), beta polypeptide 2-like 1	3.99	0.001
RPL30	ribosomal protein L30	3.91	0.043
USP9X	ubiquitin specific peptidase 9, X-linked	3.89	0.023
RAB7A	RAB7A, member RAS oncogene family	3.78	0.021
RYR3	ryanodine receptor 3	3.73	0.006
SPTB	spectrin, beta, erythrocytic	3.69	0.001
NARS	asparaginyl-tRNA synthetase	3.60	0.014
TPP2	tripeptidyl peptidase II	3.58	0.048
RAB5B	RAB5B, member RAS oncogene family	3.52	0.033
NUB1	negative regulator of ubiquitin-like proteins 1	3.33	0.047
RAB8B	RAB8B, member RAS oncogene family	3.31	0.027
ARL6IP5	ADP-ribosylation factor-like 6 interacting protein 5	3.31	0.005
ATP5O	ATP synthase, H+ transporting, mitochondrial F1 complex, O subunit	3.26	0.001
COPS7A	COP9 signalosome subunit 7A	3.12	0.008
GPX1	glutathione peroxidase 1	3.09	0.004
CANX	Calnexin	3.04	0.010
VBP1	von Hippel-Lindau binding protein 1	3.02	0.024
Cyb5r3	cytochrome b5 reductase 3	3.00	0.012
DMD	Dystrophin	2.87	0.000
PRDX3	peroxiredoxin 3	2.85	0.008
LYPLA1	lysophospholipase I	2.84	0.008
VDAC2	voltage-dependent anion channel 2	2.83	0.031
PSMD1	proteasome (prosome, macropain) 26S subunit, non-ATPase, 1	2.83	0.013
Rrbp1	ribosome binding protein 1	2.76	0.050
CTH	cystathionine gamma-lyase	2.72	0.023
PSMD2	proteasome (prosome, macropain) 26S subunit, non-ATPase, 2	2.70	0.035
GLO1	glyoxalase I	2.68	0.011
FSD2	fibronectin type III and SPRY domain containing 2	2.68	0.019
UBE2N	ubiquitin-conjugating enzyme E2N	2.66	0.010
COX4I1	cytochrome c oxidase subunit IV isoform 1	2.63	0.019
CUL3	cullin 3	2.61	0.015
GLUD1	glutamate dehydrogenase 1	2.60	0.001
DCUN1D2	DCN1, defective in cullin neddylation 1, domain containing 2	2.60	0.050
LANCL2	LanC lantibiotic synthetase component C-like 2 (bacterial)	2.58	0.059
Ktn1	kinectin 1	2.57	0.027
SGCG	sarcoglycan, gamma (35kDa dystrophin-associated glycoprotein)	2.51	0.048
CARS	cysteinyl-tRNA synthetase	2.49	0.018
RAB12	RAB12, member RAS oncogene family	2.44	0.049
UQCRC2	ubiquinol-cytochrome c reductase core protein II	2.41	0.003
VDAC1	voltage-dependent anion channel 1	2.40	0.004
EIF3B	eukaryotic translation initiation factor 3, subunit B	2.37	0.042
CISD2	CDGSH iron sulfur domain 2	2.30	0.009
OARD1	O-acyl-ADP-ribose deacylase 1	2.29	0.029
KLHL40	kelch-like family member 40	2.28	0.046
OGDH	oxoglutarate (alpha-ketoglutarate) dehydrogenase (lipoamide)	2.26	0.028
PPIB	peptidylprolyl isomerase B (cyclophilin B)	2.24	0.020
CSPG4	chondroitin sulfate proteoglycan 4	2.13	0.045
YWHAG	tyrosine 3-monooxygenase/tryptophan 5-monooxygenase activation protein, gamma	2.10	0.016
YWHAH	tyrosine 3-monooxygenase/tryptophan 5-monooxygenase activation protein, eta	2.07	0.015
TMED10	transmembrane emp24-like trafficking protein 10 (yeast)	2.06	0.007
ALDH1L2	aldehyde dehydrogenase 1 family, member L2	2.05	0.018
PSMB1	proteasome (prosome, macropain) subunit, beta type, 1	2.02	0.044
EMC2	ER membrane protein complex subunit 2	1.98	0.030
RPL12	ribosomal protein L12	1.98	0.009
HSPB2	heat shock 27kDa protein 2	1.96	0.028
DHRS7B	dehydrogenase/reductase (SDR family) member 7B	1.96	0.037
ATP2B4	ATPase, Ca++ transporting, plasma membrane 4	1.93	0.013
SPTAN1	spectrin, alpha, non-erythrocytic 1	1.86	0.031
PFKM	phosphofructokinase, muscle	1.86	0.001
MYOM2	myomesin 2	1.84	0.007
MYLK2	myosin light chain kinase 2	1.83	0.039
DDX1	DEAD (Asp-Glu-Ala-Asp) box helicase 1	1.80	0.018
PSMA5	proteasome (prosome, macropain) subunit, alpha type, 5	1.79	0.019
BAG3	BCL2-associated athanogene 3	1.76	0.034
SRL	Sarcalumenin	1.71	0.006
AMPD1	adenosine monophosphate deaminase 1	1.68	0.046
NDUFS8	NADH dehydrogenase (ubiquinone) Fe-S protein 8, 23kDa (NADH-coenzyme Q)	1.61	0.014
CACNA2D1	calcium channel, voltage-dependent, alpha 2/delta subunit 1	1.57	0.053
ANXA4	annexin A4	1.54	0.025
***Down-regulated proteins (47) in breast muscle obtained from Pedigree Male broilers exhibiting high FE*.**			
DCTN2	dynactin 2 (p50)	-11.19	0.006
UBQLN4	ubiquilin 4	-7.95	0.012
SPINK5	serine peptidase inhibitor, Kazal type 5	-7.61	0.015
BCAP29	B-cell receptor-associated protein 29	-7.24	0.018
CAPZA2	capping protein (actin filament) muscle Z-line, alpha 2	-5.93	0.038
C4BPA	complement component 4 binding protein, alpha	-5.62	0.012
EIF4G1	eukaryotic translation initiation factor 4 gamma, 1	-5.06	0.014
EDF1	endothelial differentiation-related factor 1	-3.88	0.033
ALDH9A1	aldehyde dehydrogenase 9 family, member A1	-3.85	0.029
USP15	ubiquitin specific peptidase 15	-3.77	0.026
SMYD1	SET and MYND domain containing 1	-3.74	0.028
EML1	echinoderm microtubule associated protein like 1	-3.24	0.015
PABPC1	poly(A) binding protein, cytoplasmic 1	-3.23	0.008
NAP1L1	nucleosome assembly protein 1-like 1	-3.20	0.018
ACTA1	actin, alpha 1, skeletal muscle	-2.89	0.000
UBE2V1	ubiquitin-conjugating enzyme E2 variant 1	-2.85	0.019
SOD1	superoxide dismutase 1, soluble	-2.81	0.015
PABPC4	poly(A) binding protein, cytoplasmic 4 (inducible form)	-2.76	0.009
ACTC1	actin, alpha, cardiac muscle 1	-2.71	0.000
MYBPC2	myosin binding protein C, fast type	-2.59	0.000
ALB	Albumin	-2.57	0.004
FKBP4	FK506 binding protein 4, 59kDa	-2.51	0.058
IQGAP1	IQ motif containing GTPase activating protein 1	-2.46	0.163
ACTG1	actin, gamma 1	-2.32	0.012
MYL1	myosin, light chain 1, alkali; skeletal, fast	-2.09	0.001
PEPD	peptidase D	-2.09	0.004
SARS	seryl-tRNA synthetase	-2.08	0.031
CAMK2D	calcium/calmodulin-dependent protein kinase II delta	-2.05	0.015
APOA1	apolipoprotein A-I	-2.00	0.057
PACSIN3	protein kinase C and casein kinase substrate in neurons 3	-1.81	0.011
FBP2	fructose-1,6-bisphosphatase 2	-1.77	0.008
NAP1L4	nucleosome assembly protein 1-like 4	-1.75	0.024
CCT2	cell division cycle 42	-1.67	0.010
RAB11A	RAB11A, member RAS oncogene family	-1.67	0.032
CCT2	chaperonin containing TCP1, subunit 2 (beta)	-1.64	0.002
GPI	glucose-6-phosphate isomerase	-1.59	0.043
ENO2	enolase 2 (gamma, neuronal)	-1.58	0.044
EEF1A1	eukaryotic translation elongation factor 1 alpha 1	-1.52	0.029
PBLD	phenazine biosynthesis-like protein domain containing	-1.49	0.044
LDB3	LIM domain binding 3	-1.49	0.019
EIF4A2	eukaryotic translation initiation factor 4A2	-1.49	0.015
HN1L	hematological and neurological expressed 1-like	-1.46	0.040
GAPDH	glyceraldehyde-3-phosphate dehydrogenase	-1.45	0.055
EEF1A2	eukaryotic translation elongation factor 1 alpha 2	-1.44	0.015
P4HB	prolyl 4-hydroxylase, beta polypeptide	-1.44	0.052
CKM	creatine kinase, muscle	-1.41	0.036
CAP2	CAP, adenylate cyclase-associated protein, 2 (yeast)	-1.31	0.014

#### Phenotypic impact factor (PIF) and Hierarchical cluster analysis

To identify and interpret DE proteins, we employed two complementary approaches. Firstly, we identified the edges of the MA distribution of all proteins using a modified DE called Phenotypic Impact Factor (PIF) [32], taking the liberal nominal (*P* < 0.10) approach of identifying the 5% most up regulated and 5% most down regulated proteins. Each PIF is computed by multiplying the average abundance by DE for all proteins. This has a number of appealing numerical characteristics such as tracking the edge of the MA plot distribution and de-emphasizing lowly abundant proteins which are inherently noisier as they are close to the detection limit of the technology. These 182 extreme DE-PIF proteins were assessed for functional enrichment using the two list import function in GOrilla [33] which compares the functional contents of protein lists using hypergeometric statistics.

**Fig 3:**
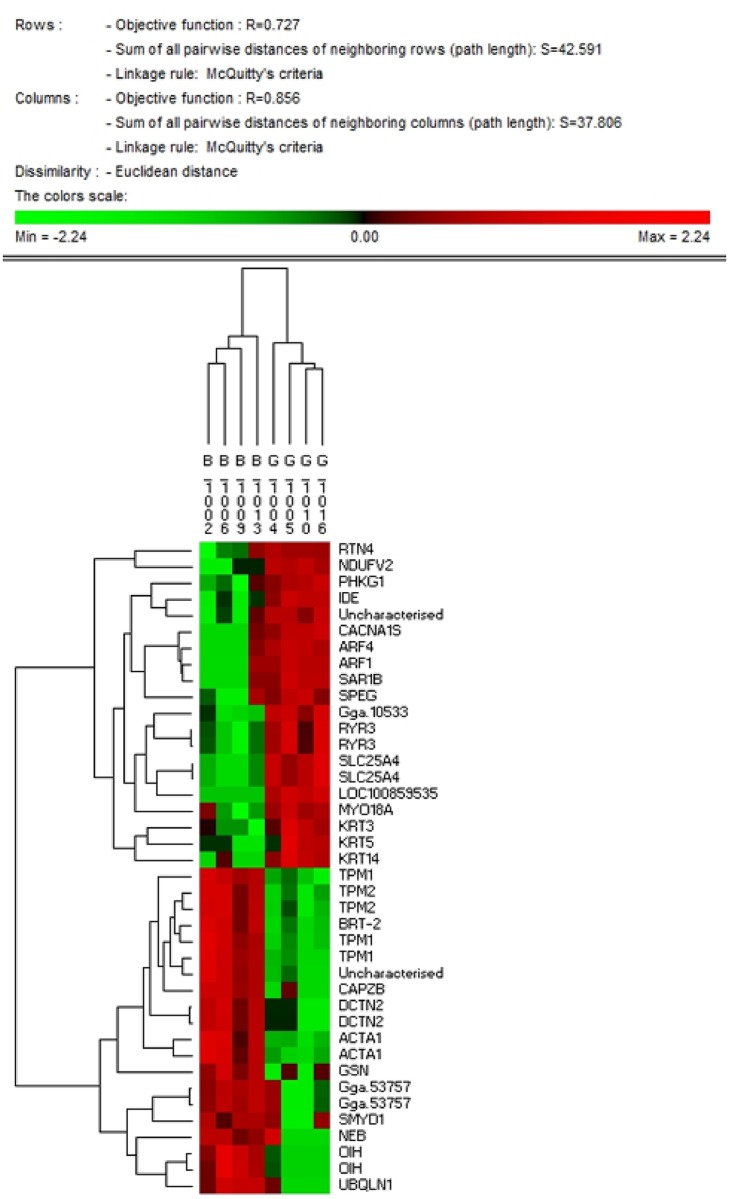
A hierarchically clustered heat map of the 40 most differentially expressed proteins as identified by PIF. Each cell contains the normalized expression of the protein at the individual bird level. Red denotes high expression, green denotes low expression. All 8 birds discriminate into their correct treatment group of origin (HFE or LFE) and relatedness within a group is also apparent. Proteins are clustered by patterns of co-expression across the 8 birds. Strong co-expression is observed for functionally related proteins e.g. ARF1 with ARF4 and KRT3 with KRT5 and KRT14 and the muscle structural proteins, namely TPM1, TPM2 and CAPZB. Different fragment of the same protein are highly co-expressed. Note: ‘B’ are birds with a low FE phenotype whereas ‘G’ are birds with a high FE phenotype. **Abbreviations:** (RTN4), reticulon 4; (NDUFV2), NADH dehydrogenase (ubiquinone) Fe-S protein 7; (PHKG1), phosphorylase kinase gamma 1; (IDE), insulin degrading enzyme; (CACNA15), calcium channel voltage dependent 1 L type, alpha 15; (ARF), ADP ribosylation factor 4 and 1; (SAR1B), SAR1-ADP ribosylation factor, (SPEG), striated muscle preferentially expressed protein; (Gga), golgi associated gamma adaptin; (RYR3) ryanodine receptor 3; (SLC26A4), adenine nucleotide translocase 1; (MYO18A), myosin 18A; (KRT), keratin; (TPM), tropomyosin; (BAT2), large proline rich protein; (CAPZB), F-actin capping protein subunit beta; (DCTN2), dynactin 2; (ACTA1), alpha actin 1; (GSN), gelsolin; (SMYD1), myosin interacting protein; (NEB), lambda protein phosphatase; (OIH) ovoinhibitor; (UBQLN), ubituilin family proteins

Next, we identified the top 40 proteins by PIF (20 up- and 20 down-regulated) using hierarchical cluster analysis. We imported the matrix with as many columns as birds (8) and as many rows as proteins (40), where each cell contains the log_2_ transformed abundance value for that protein and individual into PermutMatrix software [34], normalizing on rows. We applied hierarchical clustering on both rows and columns and exported the resulting dendrogram as a JPEG (Fig 3).

#### Bayes Methods and Ingenuity Pathway Analysis

A moderated *t*-statistic and its corresponding *P*-value was used based on empirical Bayes methods [35] for each peptide. Ingenuity Pathway Analysis (IPA; Qiagen, Valencia, CA; http://www.ingenuity.com) software was used for functional annotation of proteins, as well as canonical pathway analysis, upstream analysis, and network discovery. Upstream regulator analysis by IPA is based on prior knowledge of expected effects between transcriptional regulators and their target genes from published literature citations stored in the IPA program. The analysis determines how many known targets or regulators are within the user’s dataset, and compares the DE to the expected relationship in the literature. If the observed direction of change is mostly consistent with either activation or inhibition of the transcriptional regulator, then a prediction is made and an activation z score is generated that is also based on literature-derived regulation direction (i.e. “activating” or “inhibiting”). Activation z scores > 2.0 indicate that a molecule is activated whereas activation z scores of < -2.0 indicate that a target molecule is inhibited. Qualified predictions can also be made of high, moderate, or low absolute z scores of 1.7 or more, 1.5 to 1.7, or less than 1.5 and greater than 1.0, respectively. The p-value of overlap measures whether there is a statistically significant overlap between the dataset molecules and those regulated by an upstream regulator is calculated using Fisher’s Exact Test, and significance is attributed to p-values < 0.05.

### Quantifying the mitoproteome

To estimate mitochondrial content between the groups, we downloaded the human mitoproteome from the Mitominer website (http://mitominer.mrc-mbu.cam.ac.uk/release-3.1/begin.do). This gave us a list of 1046 nuclear- and mitochondrial-encoded mitochondrial proteins of which we identified 228 matches in our proteomics data set. We generated a proxy for tissue mitochondria content by overlaying the MA data of the mitoproteome onto the MA plot and assessing its deviation from 50:50 equilibrium by binomial statistics (Fig 2A).

## Results and Discussion

### I. Global distribution of proteins and the mitochondrial proteome

The global distribution of DE for the 1817 proteins is illustrated in Fig 2A and 2B. The plot is symmetrical and centered on 0 (for DE and protein abundance). Names of proteins that are abbreviated can be found in Tables 1 or 2 or in the figure legends. As with all MA plots, the noise is greater for low abundant proteins on the left hand side because the data approaches the detection limit of the technology, and the spread of DE data is greater. For abundance (A) values less than 4.0, there are often missing values in a subset of samples, so these DE estimates can arguably be considered less reliable. Some prominent outlier proteins have been labeled (Fig 2A) and include the mitochondrial energy transfer proteins (SLC25A4 and VDAC2) up-regulated in high FE, and the slow muscle isoforms (TPM2 and ACTC) down-regulated in high FE. The muscle fiber composition observation is consistent with an RNA sequencing analysis performed on the same samples (unpublished observations, manuscript in review) and observations of muscle whitening made in other FE selected agricultural species, such as pigs (e.g. [36]). Proteins expected to be very abundant in fast type IIB muscle fibers such as the glycolysis proteins GAPDH, PKM, and LDHA; and the fast fiber subunits MYH2 and MYOM2 are among the most abundant proteins on average across the entire data set. The CKM protein which transfers phosphate between ATP and creatine phosphate, a core energetic process in muscle cells, is also highly abundant in all samples. These results reflect the fact that broiler breast muscle is 100% type IIB fibers at a histological level [37]. The presence of some slow subunits in our data (and associated RNAseq data, not shown) reaffirms that fiber composition is more of a mosaic at the molecular level than it is at the whole fiber level.

**Table 2:** Abbreviations and names of proteins not found in Table 1 or figure legends.

Abbreviation	Protein Name
C1QBP	Complement component 1Q subcomponent binding protein
COQ9	Coenzyme Q9 (mitochondrial)
DES	Desmin
DSP	Desmoplakin
JUP	Junction plakoglobin
LDHA	Lactate Dehydrogenase A
LMB2	Lamin B2
MYOM2	Myomesin 2
MYH2	Myosin Heayy Chain 2
PKM	Pyruvate kinase (muscle)
PPA2	Pyrophosphatase (inorganic)2
PTRH2	Peptidyl tRNA hydrolase (mitochondrial)
SYMN	Synem

The extreme 10% from the entire dataset (comprising the 5% up- and 5% down-regulated PIF proteins analysis; i.e. 91 up- and 91 down-regulated, 182 PIF proteins total, See Fig 2B) in high FE were subject to functional enrichment analysis in GOrilla. The up-regulated 91 proteins were enriched for intermediate filament (FDR Q-value < 0.0001) based on the presence of DES, KRT3, KRT4, KRT5, KRT15, KRT75, LMNB2, DSP, SYNM and JUP). The down-regulated list received no significant formal enrichment by this approach

The mitoproteome of 228 proteins was significantly skewed towards the High FE birds, indicating a higher tissue mitochondria content (binomial probability *P* < 0.00001; Fig 2A). These findings are consistent with the results of global gene expression analysis (RNAseq) that was carried out on the same set of breast muscle samples as the present proteomic dataset (unpublished observations, manuscript in review). The mitochondrial proteome expression elevation was likely not the result of increased numbers of mitochondria as mitochondrial DNA (a proxy for mitochondrial numbers) was the same in both HFE and LFE samples (S2 Fig). Additionally, Kim et al. [38] provided clear evidence that mtDNA copy number or mitochondrial mass was not associated with increased mitochondrial numbers and/or increased mitochondrial protein expression in chicken embryo fibroblasts. Within the top up or down 5% of phenotype impact factors (PIF), there were 15 up-regulated mitoproteome proteins (SLC25A4, KRT5, IDE, NDUFV2, TUBA8, VDAC2, ACSL1, NDUFB5, COX4I1, UQCRFS1, NARS, PTRH2, PPA2, C1QBP, PRDX3) and only 5 down-regulated proteins (COQ9, MRPL44, ACSF2, ALDH9A1, SOD1) (Fig 2B).

In the cluster dendrogram (Fig 3), red and green represent up- and down-regulated expression in the high FE phenotype, respectively. Cluster analysis for the extreme 40 DE proteins (determined by PIF analysis) clearly discriminates the 8 birds into the FE groups they originated from. Two birds, B1013 and G1004, have a slight tendency to be outliers but are still more similar to members of their own group than to the other FE group. Among the proteins present in this nominal top 40 are mitochondrial proteins (NDUFV2, SLC25A4, IDE and KRT5), non-mitochondrial metabolism (PHKG1), and muscle sarcomeric structure (TPM1, TPM2, ACTA1 and CAPZB). As shown in Fig 3, clustering proteins by patterns of co-expression across the 8 samples produces some functional groupings of cytoskeletal keratins (KRT3, KRT5, and KRT14), muscle structural isoforms (TPM1 and TPM2), and 2 different protein isoforms of ARF (ARF1 and ARF4). Not surprisingly, different protein fragments of the same protein are always strongly co-expressed. This latter observation can be taken as a form of technical validation for our approach.

### II. Pathway Analysis

The 152 DE proteins (≥ 1.3 fold, P < 0.05) in breast muscle of high and low FE PedM broilers are presented in Table 1. The proteins in Table 1 are listed in order of the greatest to the least differentially expressed in the high FE compared to low FE phenotype. This DE ranking is related to the PIF metric output described above, but not identical because only fold difference (without multiplying by protein abundance) is reported. The DE proteins in Table 1 were organized by Ingenuity Pathway Analysis (IPA) into pathways and functional groupings, as well as predicted activation or inhibition of upstream regulators. Because of the amount of information on protein expression available in this dataset, we will focus on a) canonical pathways associated with mitochondrial function and oxidative phosphorylation, and b) selected upstream regulators.

#### Mitochondrial function—oxidative phosphorylation

**Fig 4:**
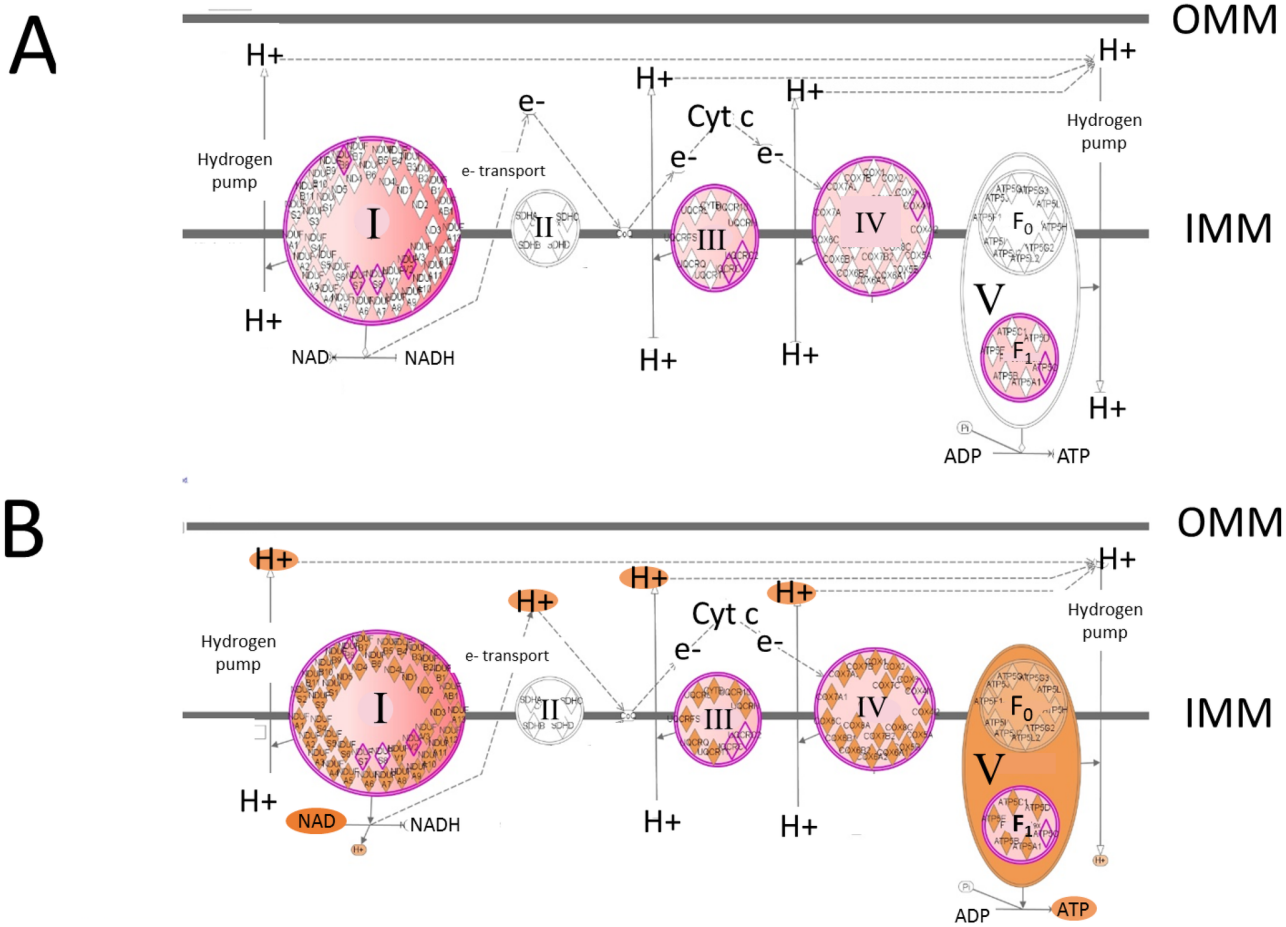
The canonical pathway of oxidative phosphorylation. The canonical pathway of oxidative phosphorylation (generated by the IPA program) showing; A) The differential expression of proteins in breast muscle associated with the electron transport chain on (Complex I, II, III, IV, and V) that were up-regulated (outlined in pink) in the high feed efficiency phenotype. Pumping of hydrogen ions (H+) creates a proton motive force between the inner (IMM) and outer (OMM) mitochondrial membranes that is used to drive ATP synthesis B) Using the molecule activity predictor function of IPA for the oxidative phosphorylation pathway, the results indicate that Complex I, III, and IV, and V in breast muscle are predicted to have greater activity (indicated by the orange color) in the high compared to the low FE phenotype.

Mitochondrial function and oxidative phosphorylation are listed as the first and fifth top canonical pathways, respectively, in the proteomic dataset (Table 3). This is consistent with the skewing of the mitoproteome towards the high FE birds as indicated above. Projection of the up-regulated mitochondrial proteins in the high FE phenotype (from Table 1) onto the canonical pathway of oxidative phosphorylation is presented in Fig 4A. From these data, the IPA program predicted that Complex I, III, IV and V activities would be greater (shown in orange in Fig 4B) in breast muscle of the high FE compared to the low FE phenotype in this study. The IPA program also predicted that Complex II would be activated in the high FE phenotype using a 1.2 fold expression cutoff (*P <*0.05, data not shown). These predicted increases in respiratory chain activities are in agreement with the general increase in mitoproteome expression discussed previously. The predicted increases in complex activities also concurs with results from enzymatic analysis conducted previously on muscle, liver and duodenal tissue obtained from the same pedigree male broiler line indicated that there was a general increase in complex activities in the high compared to low FE phenotype [10]. Similarly, complex activities were higher in the lumbar multifidus (LM) muscle of Ghezel male lambs with low RFI (more feed efficient) compared to those with high RFI (less efficient) [39]. We hypothesized that the lower complex activities in the low FE phenotype were due to a pervasive protein oxidation from mitochondrial ROS production observed in tissue homogenate and mitochondrial fractions [9, 10].

**Table 3:** Top canonical pathways. The top 6 canonical pathways (based on p value) generated by Ingenuity Pathway Analysis based on differentially expressed proteins in breast muscle obtained from broiler breeder males.

Canonical Pathway	p Value
Mitochondrial Dysfunction	4.85E-09
Glycolysis I	9.24E-07
Caveolar-mediated Endocytosis Signaling	9.62E-07
Clathrin-mediated Endocytosis Signaling	1.10E-06
Oxidative Phosphorylation	1.38E-06
Protein Ubiquitination	2.87E-06

Two up-regulated proteins in the high FE breast muscle integral to mitochondrial function are adenine nucleotide translocase (ANT or SLC25A4) and voltage dependent anion channel 1 and 2 (VDAC1 and VDAC2) (Table 1). ANT and VDAC2 were also identified as PIF molecules as described above. With the exception of a few lipophilic compounds (e.g. acetaldehydes, short chain fatty acids and molecular oxygen) all metabolites that enter or leave mitochondria must pass through the VDAC channel in the outer mitochondrial membrane [40]. The channels play an important role in transmitting redox information between the mitochondria and nucleus [41] in part by the ability of superoxide to move out of the mitochondria through VDAC channels [42]. The combined activities of ANT and VDAC are responsible for shuttling ADP and ATP between the mitochondrial matrix and the cytosol thus facilitating cellular ‘work’ while providing substrate for ATP synthase that uses proton motive force generated by the respiratory chain to produce ATP from ADP and Pi.

The ANT-VDAC coupling in conjunction with mitochondrial creatine kinase (mtCK) facilitates the transfer of high energy phosphate bonds from ATP to creatine to form phosphocreatine (see review [43]). In the model presented by Schlattner et al. [43], mtCK, located in the intramembranous space, has contact sites with both VDAC on the outer membrane and ANT on the inner mitochondrial membrane. This arrangement facilitates direct channeling of ATP to mtCK that catalyzes the transfer of phosphate groups to creatine to form phosphocreatine that is released to the cytosol. A large cytosolic pool of phosphocreatine can ‘….*then be used as a temporal buffer to maintain constant global and local ATP/ADP ratios over a wide range of workloads…and spatial energy buffering; i*.*e*. *for an energy shuttle between ATP-providing or-consuming processes*.” [43].

There are at least four subunit isoforms of CK expressed in vertebrate tissues: two cytosolic forms, CKM (muscle) and CKB (brain), and two mitochondrial (mt) CK isoforms. In vivo, CKM and CKB isoforms combine to provide three typical dimeric cytosolic isozymes: MM-, MB- and BB-CK [44]. In the proteomics dataset, CKB was up regulated 8.9 fold whereas CKM was down regulated -1.47 fold (Table 1) compared to the low FE phenotype; the mtCK isoform was not detected. Quest et al [45] suggested that tissue-specific dimerization of B-CK monomers in chicken tissues could represent a mechanism for specifying the intracellular distribution of this cytosolic isoform that in turn could influence energy transfer by phosphocreatine. How this differential expression of CKM and CKB relates to energy transduction in the high and low FE phenotypes is not apparent at this time, but further studies are warranted. We have also observed that mitochondrial CK (*CKMT1A)* was up-regulated in high FE in the RNA sequencing data being analyzed concurrently (manuscript in preparation).

Another up-regulated mitochondrial protein in the high FE phenotype was peroxiredoxin III (PRDX3, 2.85 fold, Table 1). In mammals, PRDX3 is located exclusively in the mitochondria and functions like a GSH peroxidase in converting hydrogen peroxide to water [46–48] and maintains mitochondrial mass and membrane potential [49]. Increased PRDX3 expression in high FE mitochondria could therefore contribute to lower mitochondrial ROS production and lower protein oxidation [10] and higher expression of electron transport chain proteins in the present study (Fig 4).

The increased expression of mitochondrial electron transport chain proteins shown in Fig 4 contrasts with recent reports in 1) female Large White pigs (115 kg, from lines divergently selected for high or low RFI [36]), and 2) in the top 5 and bottom 5 of a group of 238 castrated Yorkshire purebred boars individually phenotyped for RFI [50]. Using a cDNA microarray, Vincent et al. [36] reported that mRNA expression of genes encoding for protein subunits for Complex I (ND1, ND2, ND4, NDUFB9), and isocitrate dehydrogenase, were down-regulated in the low RFI pig. It is also contrary to the reduction in slow subunits (TPM2 and ACTC) we observed in the high FE birds [5, 6], as it is well established across all species that slow, aerobic fibers have a higher mitochondrial content than fast, glycolytic fibers. Vincent et al. [36] also reported lower ATP5A1 (a subunit of Complex V, ATP synthase) and, in contrast to what was mentioned above, lower mitochondrial CK expression in the low RFI females compared to the high RFI females. There are at least three possible explanations for differences between the present study and Vincent et al. [36]; 1) species difference (swine vs broiler), 2) sex (female vs. male), and 3) protein expression does not always follow gene expression differences. With respect to point 3 above, projection of the DE genes in the cDNA microarray dataset from Kong et al. [5] onto the oxidative phosphorylation canonical pathway using the overlay function in IPA, revealed up-regulation of genes and prediction of activation of Complex I but down-regulation of genes and prediction of inhibition of Complex III and IV activities (data not shown).

Using qPCR, Jing et al. [50] reported decreased mRNA expression of 16 of 17 mitochondrial DNA encoded proteins in low RFI castrated boars including four from Complex I and one from Complex III along with decreased expression of the 5’AMP activated protein kinase gamma 2 (AMPKγ2) and peroxisome proliferator-activated receptor gamma coactivator 1-alpha (PPARPGC1A, or PGC1-α. PGC1-α is a master regulator of mitochondrial biogenesis [51–53] and AMPK is an energy sensing molecule that responds to increased AMP to ATP levels by increasing energy production (e.g. fatty acid β oxidation, glycolysis, oxidative phosphorylation) and decreasing energy consumption reactions (e.g. gluconeogenesis, fatty acid synthesis) [54–57]. Thus, the decreased gene expression of mitochondrial DNA-encoded proteins reported by Jing et al. [50] is congruent with the decreased expression of AMPK and PGC1-α. Similarly, increased expression of respiratory chain proteins in the current study is congruent with increased AMPK mRNA expression in the high FE broiler [6] as well as the predicted activation of PRKAA2 (AMPKα2, catalytic subunit of AMPK), and moderate activation of PPARGC1α (PGC1-α) derived from the proteomic dataset discussed below. However, a fundamental difference (besides species) between Jing et al [50] and the current proteomics study is the use of castrated males vs. intact males in the present study. Broilers typically begin to show sexual dimorphism (increased comb and wattle size and heavier body weights) around 4 to 5 wk of age; two weeks prior to when FE phenotyping occurred in our FE model. Shifts in steroid hormone levels between castrated and intact males could have a number of effects on nuclear and mitochondrial gene and protein expression; especially since thyroid and steroid hormone receptors are found in mitochondria as well as the nucleus [58].

#### Upstream Regulators

The IPA program was used to predict activity of upstream regulators based on DE proteins in the high and low FE phenotypes. The predictions are based on activation z-score and *P*-value of overlap generated from literature based citations for each differentially expressed molecule/protein. A *P* overlap value of < 0.05 and an activation z-score of > 2.0 or < -2.0 indicates that a specific molecule is predicted to be activated or inhibited, respectively. It is also possible to provide qualified predictions of activation or inhibition classified as strong, moderate, or weak that is based on a combination of the activation z-score and *P*-value of overlap. A list of differentially expressed proteins upon which the prediction was based (shown in red and green letter for up-regulated and down-regulated proteins in the high FE phenotype, respectively) is provided in Table 4.

**Table 4:** Upstream regulators predicted to be activated or inhibited in the proteomic dataset.

Upstream Regulator^2^	Predicted Activity (In HFE)	Activation z-score	p-value of overlap	Target molecules used in predictions: No underline: up-regulated in low FE Underline: up-regulated in high FE (Names of proteins corresponding to abbreviations can be obtained from Tables 1 & 2)
INSR	Activated	2.425	4.29E-05	ACTA1, GAPDH, TP5O, COX4I1, FABP4, MYOM2, NDUFV2, OGDH, UQCRC1, UQCRC2
NFE2L2	Activated	2.382	1.54E-06	ACTG1, SOD1, RF1, EIF4G2, GNB2L1, GPX1, IDE, NARS, PPIB, PSMA5, PSMB1, PSMD1, RYR3,
IGF1R	Activated	2.236	2.95E-04	ACTA1, ATP5O, COX4I1, MYOM2, NDUFV2, UQCRC1, UQCRC2
progesterone	+++	1.954	4.55E-02	ACTA1, CAV1, IDE, KRT15, RAP1B, RYR3, YWHAG
PRKAA2	+++	1.951	3.20E-03	ACTA1, ALB, P4HB,COX4I1,
T_3_	+++	1.914	1.15E-03	ACTA1, ACTC1, ALB, APOA1, COX4I1, CTH, FABP4, PRDX3
PPARA	+	1.571	1.18E-03	ACTA1, ALDH9A1, APOA1, PBLD, COX4I1, CTH, FABP4, GLUD1, UQCRC1
PPARGC1A	+	1.531	4.37E-05	ATP5O, COX4I1, FABP4, GPX1, NDUFV2, PRDX3, SOD1
SRF	---	-1.961	1.49E-03	ACTA1, ACTC1, CKM, LBD3, MYL1, DMD, GLRX3
MAP4K4	Inhibited	-2.000	6.85E-03	NDUFS8, OGDH, PFKM, UQCRC1
RICTOR	Inhibited	-3.873	7.58E-11	ATP5O, COX4I1, NDUFB8, NDUFS7, NDUFS8, NDUFV2, PPA2, PSMA5, PSMB, PSMD1, PSMD2, RPL12, RPL30, UQCRC1, UQCRC2

^1^ Prediction of activity of upstream regulators (positive or negative) based on activation z scores and p value of overlap (See Materials and Methods). Classification of activities are shown as predicted significantly strong positive or predicted strong negative based on a activation z score of > 2 and <−2, respectively. Qualified classifications of strong, moderate, or weak activation are indicated by +++, ++, +, respectively and qualified classification of strong inhibition is indicated by—.

^2^ Abbreviations: INSR, insulin receptor; IGF1R, insulin like growth receptor 1; NFE2L2, nuclear factor, erythroid 2-like 2; AMPKα, AMP activated protein kinase (α subunit), progesterone; T_3_, triiodothyronine; PPARA, peroxisome proliferator-activated receptor; PPARGC1A, peroxisome proliferator-activated receptor gamma coactivator 1-alpha; RICTOR, rapamycin independent companion of target of rapamycin; MAP4K4, mitogen-activated protein kinase kinase kinase kinase 4; SRF, serum response factor.

#### A. Activated Upstream Regulators

1. Insulin receptor (INSR), and insulin-like growth factor 1 receptor (IGF1R). Based on expression of downstream targets, INSR and IGF1R were predicted to be activated in the breast muscle from the high FE phenotype (Table 4). A thorough review of the insulin receptor signaling pathway in birds has presented differences that exist between mammals and birds [59]. After binding to its receptor, insulin activates INSR tyrosine kinase activity and phosphorylates downstream signaling molecules that includes the insulin receptor substrate (IRS) family leading to glucose homeostasis and energy storage [60–64]. Insulin signaling is dependent upon endocytosis of the receptor that occurs in conjunction with caveolae and caveolin proteins (particularly caveolin 1, CAV-1) on the surface membrane of target cells [65]. In this regard, the 9.4 fold up-regulation of CAV-1[6] (Table 1), which is specific for insulin in mammals [65] would facilitate insulin receptor signaling in the high FE phenotype. Caveolae-mediated endocytosis signaling was the third ranked canonical pathway in the proteomics dataset (Table 3).

As in mammals, insulin increases glucose uptake in chicken muscle [66]. However, this is mediated by glucose transporter 8 (Glut-8) which is the avian analog to mammalian Glut-4 [67, 68]. A well characterized component of insulin receptor signaling is increased protein synthesis mediated by PI3K-Akt and mTOR (mammalian target of rapamycin) pathways (see Fig 27.3; in Dupont et al. [59]). Insulin-like growth factors (IGFs) along with PI3K/Akt signaling regulate muscle growth in various organisms (e.g. [69, 70]) including chickens [71]. Insulin-like growth factor 2 (IGF2) was up-regulated in high FE commercial broiler breast muscle [4] and in the latissimus dorsi (LD) muscle of castrated pigs [50] which supports a role of insulin-like growth factor in feed efficiency. Chen et al. [72] reported that mRNA expression of IGF binding protein 3 (IGFBP3) was higher in liver of more efficient low RFI angus bull calves (progeny of third generation of selection) and indicated that this lower expression likely contributed to lower fat deposition compared to high RFI bull calves. Expression of several genes of the PI3K signaling pathway, including PI3K, p85, pIK3R1, PP2A, MAP2K4, Ras, and Jnk, were up-regulated in the high FE breast muscle tissue [6] and led us to hypothesize that mTOR played a role in the phenotypic expression of FE [73]. Additionally, Lee et al. [3] reported that several genes in the mTOR pathway were up-regulated in liver and duodenum of broilers with low RFI (i.e. higher efficiency). The results of the present study along with Zhou et al. [4] and Lee et al. [3] indicate that insulin signaling and mTOR pathways may be biomarker candidates for FE selection. There is precedent for involvement of insulin signaling in FE in cattle and swine, particularly in young animals as recently reviewed by Davis et al. [74].

Downstream target proteins of INSR and IGF1R in Table 4 include five electron transport chain proteins; ATP50, COX4I1, NDUV2, UQCRC1 and UQCRC2. Insulin has been recognized to affect mitochondrial function early on in mitochondrial physiology investigations (e.g. [75–77]. Insulin signaling is integral to electron transport chain activity by maintaining the NAD+/NADH ratio that sustains signaling for mitochondrial biogenesis mediated by PGC1-α (see review, [78]). The PGC1-α protein had a moderate (1.531) activation z-score in high FE muscle (Table 4, PPARGC1α). AMP activated protein kinase (AMPK), which acts as an energetic sensor by detecting changes in the AMP/ATP ratio in the cell, stimulates mitochondrial energy production and mitochondrial biogenesis and is also tied to the insulin signaling pathway [78]. In this regard, the catalytic subunit of AMPK (PRKAA2, or AMPKα2) was predicted to be activated (1.951 activation z-score, Table 4) and mRNA expression of AMPK and two subunits (AMPKα and AMPKγ) were elevated in the high FE phenotype [6]. In addition, mTOR mRNA expression was elevated in PedM broiler males and in male quail selected for high FE [79].

In the present study, insulin degrading enzyme (IDE) is the third highest up-regulated protein in high FE breast muscle (11.7 fold, Table 1) and was also present in the 91 proteins deemed upregulated by the PIF metric described previously. This data concurs with up-regulated IDE mRNA expression (1.4 fold) in the high FE phenotype in the cDNA microarray data (See [5]) deposited in Gene Expression Omnibus (GEO; accession number: GSE24963; http://www.ncbi.nlm.nih.gov/geo). IDE is involved in insulin signal transduction and IDE-mediated degradation of insulin has been linked to inhibition of protein degradation via a proteosomal mechanism [80–84]. Tundo et al. [85] reported that IDE also possesses heat-shock protein activity; thus IDE may also have protein chaperoning and scaffolding activities. Interestingly, Leissring et al. [86] reported a mitochondrial IDE isoform that could be instrumental in optimizing mitochondrial function. Mitochondrial proteins that are encoded by nuclear DNA typically contain a targeting sequence that must be cleaved upon entry into the mitochondria in order to be functional. Leissring et al. [86] provided evidence that IDE was capable of cleaving mitochondrial leader peptides that would play an important role in promoting optimal mitochondrial protein structure and subsequently support optimal mitochondrial function.

2. Nuclear factor, erythroid 2-like 2 (NFE2L2). Expression of the transcription factor NFE2L2 is increased in response to oxidative stress and enhances antioxidant protection by modifying genes with the antioxidant response element in their promotor regions (e.g. [87, 88]). From previous findings of higher mitochondrial ROS production and pervasive protein oxidation [8, 10], we anticipated that NFE2L2 would be activated in the low FE PedM. However, NFE2L2 was activated in the high FE PedM (Table 1), which concurs with findings in commercial broilers reported by Zhou et al. [4].

There are differences between the molecules that were used by the IPA program in the predicted activation of NFE2L2 between the present study using PedM broilers and that of Zhou et al. [4] conducted with commercial broilers. In the present study, of the downstream molecules used in predicting NFE2L2 activation (Table 4), only two antioxidant proteins were differentially expressed; glutathione peroxidase (GPX1, cytosolic) and superoxide dismutase (SOD1) that were up- and down-regulated in the high FE phenotype, respectively. As indicated above, PRDX3 was up-regulated in high FE muscle, but was not included in the downstream molecules used in predicting NFE2L2 activation. In the commercial male broiler FE model [4], up-regulated antioxidants in the high FE included 3 glutathione transferase enzymes, heme oxygenase 1, extracellular superoxide dismutase (SOD3), and thioredoxin (TXN). Whereas the ryanodine receptor (RYR3) was down-regulated in the high FE commercial broiler [4], RYR3 protein expression was up-regulated in high FE pedigree males in the present study. In addition, IDE, three proteosomal proteins (PSMA5, PSMB1, and PSMD1), ADP ribosylation factor 1 (ARF1), and NARS (asparaginyl-tRNA synthetase), (target molecules used in the prediction of NFE2L2 activation [Table 4]), did not appear in the predicted activation of NFE2L2 in high FE commercial broilers [4]. Higher expression of IDE could serve to regulate the insulin signaling and play a role in importing proteins into the mitochondria as discussed previously. Enhanced proteosomal activity in high FE seems counterintuitive for promoting greater efficiency, yet we observed higher mRNA expression of 19S proteasome in high FE breast muscle [73]. In addition, UBQLN1, another proteosomal protein, is down regulated in high FE (Fig 3) so the evidence for enhanced proteosomal activity in high FE requires further investigation.

Increased ARF1 expression in the high FE PedM broiler is of particular interest to us as this molecule was recently reported to play a critical role in mitochondrial homeostasis [89, 90]. Using inhibitory RNA to deplete ARF1, it was shown that muscle cells in *C*. *elegans* displayed altered mitochondrial morphology and diminished function [89]. The interaction of ARF1 with mitochondria enables the *‘…recruitment of a degradation machinery to mitochondria to remove toxic mitofusin/Fzo1 clusters; and it allows the extension of autophagy sequestration membranes needed for mitophagy to clear damaged mitochondria*.” [90]. Thus, increased ARF1 expression could help in maintaining overall mitochondrial function in cells by removing mitochondria, or parts of mitochondria, that are under performing. Elevated ARF1 expression in high FE PedM broiler muscle represents an additional link between mitochondria and FE. Interestingly, cluster analysis showed ARF4 to be highly co-expressed with ARF1 across the 8 samples (Fig 3), implying the possibility of shared function between the two proteins. Indeed, Nakai et al. [91] found ARF1 and ARF4 are collectively required for the integrity of recycling endosomes, with endosomes playing a role in lysosomal degradation.

Zhou et al. [4] indicated that the oxidative stress in the high FE commercial broiler was due to muscle development; i.e. that higher NFE2L2 activity was in response to inflammatory-mediated oxidative stress as there was up-regulation of a number of myokines and cytokines in their RNAseq dataset. This contrasts with the evidence of lower oxidative stress in high FE PedM broilers [10]. Additional evidence of higher stress in the low FE pedigree male broiler included the up-regulation of corticotropin releasing hormone, actin polymerization and actin-myosin stress fiber formation, heat shock proteins, and suppressor of cytokine signaling (SOC3) [5–7]. As body weights were similar between the report by Zhou et al. [4] and the present study, differences in oxidative stress cannot be explained by differences in growth rate between the studies. This leads us to hypothesize that the predicted activation of NFE2L2 in the high FE PedM broiler may play a role in directing cellular processes to occur, rather than responding to oxidative stress as indicated by Zhou et al. [4] in commercial broilers.

3. Progesterone and Triiodothyronine. Progesterone and triiodothyronine (T_3_) were predicted to be activated (z-activation scores of 1.954 and 1.951, respectively) in breast muscle of the high FE PedM broiler phenotype (Table 4). Implants containing progesterone and estrogen have been used for years to improve growth and FE in the beef industry (see review by Preston, [92]). These implants mediate their effects by stimulating growth hormone activity and the insulin/insulin- like growth factor signaling pathway (e.g. [93–95]). Thus, activation of progesterone could contribute to insulin receptor activation described above. In a separate but concurrent RNA sequencing study on the same breast muscle tissue samples, we have additional indication that progesterone is likely to be centrally involved in activation of several transcription factors in the high FE phenotype (unpublished observations).

The predicted activation of triiodothyronine (T_3_, Table 4) follows the increase in the mitoproteome in high FE but is surprising since it has long been associated with increased energy expenditure through increased basal metabolic rate. However, a report by Clement et al. [96] investigating the impact of thyroid hormone on human skeletal muscle in vivo has several remarkable parallels to the present proteomic investigation. Clement et al. [96] reported that expression of 15 genes encoding mitochondrial electron transport chain proteins were up-regulated by thyroid hormone (75 μg/day for 14 d) similar to the up-regulation of electron transport proteins shown in high FE (Fig 1). From both *in vivo* and *in vitro* studies, Enriquez et al. (1999) reported that mitochondrial RNA synthesis was directly stimulated by thyroid hormone. Based on findings of Enriquez et al. [97] and others, Psarra and Sekeris [58] indicated that the mitochondrial thyroid hormone receptor “…*is a mitochondrial*, *thyroid hormone activated transcription factor which in analogy to the action of the nuclear transcription factors*, *would exert its modulatory effect by interaction with the mitochondrial transcriptome*.*”* Clement et al. [96] also reported that several proteasome and ubiquitin-related genes were up-regulated, which concurs with up-regulated 19S proteosome mRNA expression in the high FE phenotype [73] and proteosomal proteins in the present study (see target molecules for NFE2L2, Table 4). Recently, Piekarski et al. [79] reported that gene expression of key members of the autophagy pathway were higher in the high FE phenotype, which agrees with the up-regulation of Beclin associated with thyroid hormone reported by [96]. These anecdotal similarities do not prove that thyroid hormone is involved in the phenotypic expression of FE, but do warrant further investigation.

Cystathionine gamma lyase (CTH) is also a downstream target molecule of T_3_ (Table 4). As this enzyme is involved in the transsulfuration pathway in the conversion of methionine to cysteine, it conceivably could play a role in maintaining glutathione, which is the major intracellular antioxidant, as cysteine is the rate limiting amino acid of glutathione synthesis [98].

#### B. Inhibited Upstream Regulators

1. Rapamycin independent companion of target of rapamycin (RICTOR). We previously reported that there was decreased mRNA expression of numerous actin-myosin cytoskeletal fibers in the high FE phenotype [5, 6]. RICTOR plays an important role in actin-cytoskeletal formation as impaired formation was observed in RICTOR knockout mice [99, 100]. Thus, inhibition of RICTOR in the high FE PedM broiler phenotype could play a role in the lower cytoskeletal fiber gene expression that we reported previously [5, 6]. Inhibition of serum response factor (SRF) (see below), could also contribute to lower cytoskeletal organization observed in the high FE PedM broiler. [101] reported that the RICTOR component of mTORC2 is integral in actin-stress fiber development that stimulates bone marrow derived mesenchymal stem cells to become osteoblasts. Thus, the observation that actin stress fiber formation was down-regulated in the high FE phenotype [6] could be directly related to lower RICTOR activity. Unlike ablation of regulatory associated protein of mTOR, Complex 1 (RAPTOR) activity of mTORC1, which is critical for muscle function, decreased RICTOR activity has no effect on muscle function [102].

The predicted inhibition of RICTOR, however, is incongruent with insulin signaling described above. Insulin stimulation of glucose uptake in muscle of mammals is carried out by mTORC2-mediated facilitation of glucose transport 4 (Glut-4) receptors [103]. Kumar et al. [104] reported that there was a decrease in insulin-mediated glucose uptake and an increase in basal glycogen synthase activity in male adult mTORC2 knockout mice. To our knowledge, effects of mTORC2 on the presumed glucose transporter protein (Glut-8) and glycogen synthase activity has not been reported in avian species.

2. Mitogen-activated protein kinase kinase kinase kinase 4 (MAP4K4). In the current study, MAP4K4 was predicted to be inhibited in the high FE PedM broiler phenotype (Table 4). Guntur et al. [105] reported that MAP4K4 acts as a negative regulator of PPARγ-mediated stimulation of translation in adipocytes by suppressing the mTOR signaling pathway. More recently, Wang et al. [106] demonstrated that myotube formation was enhanced in MAP4K4 mutant C2C12 cells via a myogenic factor 5 (Myf5)-dependent mechanism and by other mitogen-activated protein kinase (MAPK)-signaling pathways leading the researchers to conclude that MAP4K4 was a novel suppressor of skeletal muscle differentiation [106]. Thus, inhibition of MAP4K4 activity could contribute to muscle development in the high FE PedM broiler muscle in the present study.

3. Serum Response Factor (SRF). Serum response factor (SRF) was predicted to be inhibited in the high FE phenotype muscle (Table 4). SRF signaling is stimulated by MAPK or RhoA pathways that enhance nuclear transcription primarily of cytoarchitecture-related genes [107]. SRF was reported to bind to the promoter region of several cytoskeletal-contractile genes in fully differentiated muscle tissue [108–110]. Miano et al. [111] reviewed the literature on SRF and presented a list of ~120 cytoskeletal-contractile genes stimulated by SRF-mediated transcription (see Table 2 on p. C73 [111]). We hypothesized that the higher degree of cytoskeletal organization would contribute to the phenotypic expression of low FE due to the increased energy expenditure needed to maintain this cellular architecture [5, 6]. From the GEO database that was deposited with this microarray set, expression of 27 genes (approximately 25% of the SRF target genes listed in Table 1 of [111]) were DE in the cDNA microarray dataset; of these 27 genes, 25 were down-regulated in the high FE phenotype (from [5]) which concurs with the predicted inhibition of SRF (and its effect on cytoarchitecture gene transcription) in the current proteomic dataset. Also, SRF mRNA expression was down-regulated 1.25 fold in the high FE phenotype (unreported observation, [5]).

### III. Summary and Conclusions

**Fig 5:**
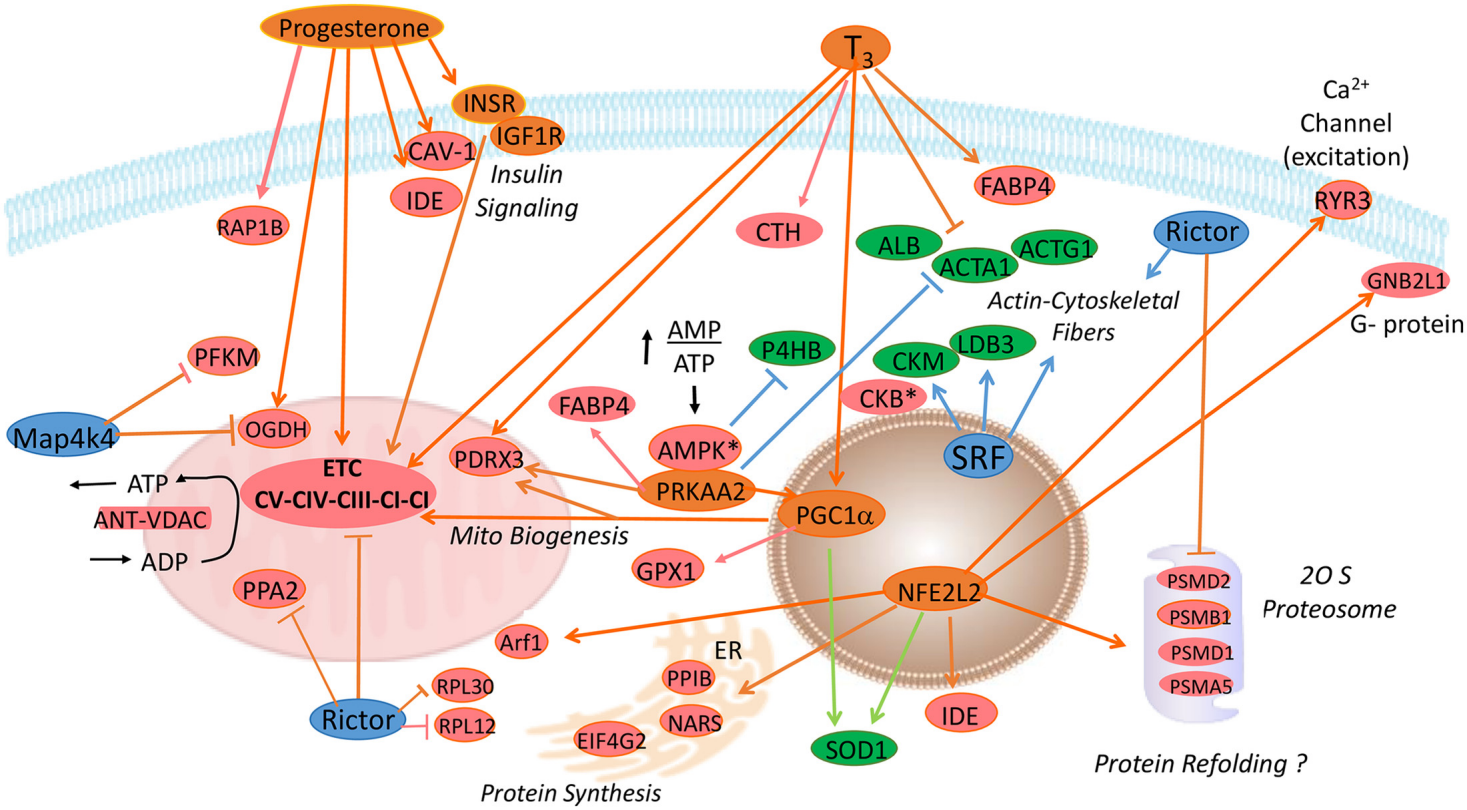
Summary of upstream regulator analysis obtained in the proteomic dataset. Upstream regulators predicted to be activated in breast muscle (based on expression of downstream molecules) of high FE pedigree broilers shown in orange included progesterone, triiodothyronine (T_3_), insulin receptor (INSR), insulin-like growth factor receptor 1 (IGF1R), peroxisome proliferator-activated receptor alpha (PPARA), peroxisome proliferator-activated receptor alpha gamma coactivator 1-alpha (PGC1α) and nuclear factor, erythroid 2-like 2 (NFE2L2). Upstream regulators predicted to be inhibited in breast muscle of the high FE phenotype shown in blue include rapamycin-insensitive companion of target of rapamycin (RICTOR), serum response factor (SRF), and mitogen-activated protein kinase 4 (MAP4K4). Orange and blue arrows indicate activation or inhibition, respectively of downstream targets. Orange lines with a bar and the end indicate the downstream target molecule would be inhibited or down-regulated in the high FE phenotype. Blue lines with a bar at the end indicate that inhibition of downstream targets would be down-regulated or diminished in the high FE phenotype. Light green arrows indicate that the expression of a downstream molecule is inconsistent with literature based results in the Ingenuity Pathway Analysis program. Proteins up-regulated in the high FE phenotype are shown in red whereas down-regulated proteins are shown in green with the complete name and fold difference in expression shown in Table 1.

The diagram shown in Fig 5 presents a summary of the results that are presented in this shotgun proteomic study conducted in a pedigree male (PedM) broiler line exhibiting high or low FE phenotypes. The results of previous studies conducted using this PedM broiler line that indicated that the high FE phenotype had better respiratory chain coupling and increased respiratory chain complex activity [8, 10], appears to be recapitulated in the proteomic analysis indicating that 4 of 5 electron transport chain complexes were predicted to be activated in the high FE pedigree broiler male (Fig 4B) and the entire mitoproteome is skewed towards the high FE birds (Fig 2A). Of considerable interest are the predictions that both progesterone and thyroid hormone would be activated in the high FE phenotype. It is well known that exogenous treatment with progesterone (as one part of a combination hormone growth promotant treatment) plays a role in improving FE in cattle that is mediated by the insulin signaling pathway. With the exception of NFE2L2 and SRF, all other upstream regulators had at least one mitochondrial electron transport protein as a target molecule that was used in their respective predictions (see Table 4). In particular, the increase in mitochondrial protein expression would be supported by moderate activation predicted for two transcription factors, PPARGC1α and PPARα, associated with mitochondrial biogenesis. With the lower oxidative stress that we have consistently observed in the high FE phenotype (e.g. [10]), we hypothesize that the predicted activation of NFE2L2 (which is typically increased in response to oxidative stress), reflects an inherent difference that helps orchestrate gene expression in the high FE phenotype. The predicted activation of SRF might be important in enhanced cytoarchitectural gene expression reported in the low FE PedM broiler phenotype [5, 8]. We believe that further analysis of the proteomic and genomic datasets that we have obtained in the high and low FE PedM broilers will generate hypotheses that can then be mechanistically tested both *in vivo* and *in vitro* to yield further insights into fundamental understanding of the cellular basis of feed efficiency.

The broad findings of the current study indicate that upstream analysis implicate involvement of progesterone and triiodothyronine that would increase expression of mitochondrial proteins as indicated in Fig 2. This would be complemented by PGC1α and PPARA that would stimulate mitochondrial biogenesis. The predicted activation of NFE2L2 would enhance proteosomal activity (in agreement with Bottje et al. [2014]) and could enhance mechanisms associated with protein refolding. NFE2L2 activation was also attributed to increased expression of several proteins (PPIB, EIF4G2, and PPIB) as well as decreased inhibition exerted by RICTOR that collectively could result in increased protein synthesis. NFE2L2 predicted activation was also based on increased expression of ARF1 (ADP ribosylation factor 1) that may help in maintaining mitochondrial homeostasis [89, 90]. Increased expression of ANT and VDAC in the inner and outer mitochondrial membranes would be instrumental in exchange of ATP and ADP to and from the cytosol.

## Supporting Information

S1 Fig Fold expression values of house-keeping proteins were similar for Low (green bar) and high FE (red bar) resulting from normalization procedures (see text for details). Proteins include: AGTN2 (alpha-actin 2), MYH1G (myosin heavy chain 1 gamma), MYH7B (myosin heavy chain 7 beta), TBB5 (tubulin beta 5 chain), TBB7 (tubulin beta 7 chain), TBA1 (tubulin alpha 1 chain). Values represent the mean ± SE (n = 4).
(TIF)

S2 Fig Polymerase chain reaction (PCR) results for determining mitochondrial (mt) DNA content in breast muscle obtained from Pedigree Male Broilers exhibiting for low or high feed efficiency phenotypes (LFE and HFE). Values represent mean ± SE (n = 4). Primer information:
mtDNA-D loop Forward—ACACCTGCGTTGCGTCCTA; Reverse—ACGCAAACCGTCTCATCGA; 18S rRNA gene Forward—TCCCCTCCCGTTACTTGGAT; Reverse—GCGCTCGTCGGCATGTA.
(TIF)
