# Correction: Proteomics of Breast Muscle Tissue Associated with the Phenotypic Expression of Feed Efficiency within a Pedigree Male Broiler Line: I. Highlight on Mitochondria

**DOI:** 10.1371/journal.pone.0159897

**Published:** 2016-07-19

**Authors:** Byung-Whi Kong, Kentu Lassiter, Alissa Piekarski-Welsher, Sami Dridi, Antonio Reverter, Nicholas James Hudson, Walter Gay Bottje

The fifth author’s name is spelled incorrectly. The correct name is: Antonio Reverter. The correct citation is: Kong B-W, Lassiter K, Piekarski-Welsher A, Dridi S, Reverter A, Hudson NJ, et al. (2016) Proteomics of Breast Muscle Tissue Associated with the Phenotypic Expression of Feed Efficiency within a Pedigree Male Broiler Line: I. Highlight on Mitochondria. PLoS ONE 11(5): e0155679. doi:10.1371/journal.pone.0155679
