# Supplementary figures and images for: Proteomics of Breast Muscle Tissue Associated with the Phenotypic Expression of Feed Efficiency within a Pedigree Male Broiler Line: I. Highlight on Mitochondria
Source: PLoS One. 2016 May 31;11(5):e0155679. doi: 10.1371/journal.pone.0155679 (PMC4887024; doi:10.1371/journal.pone.0155679)

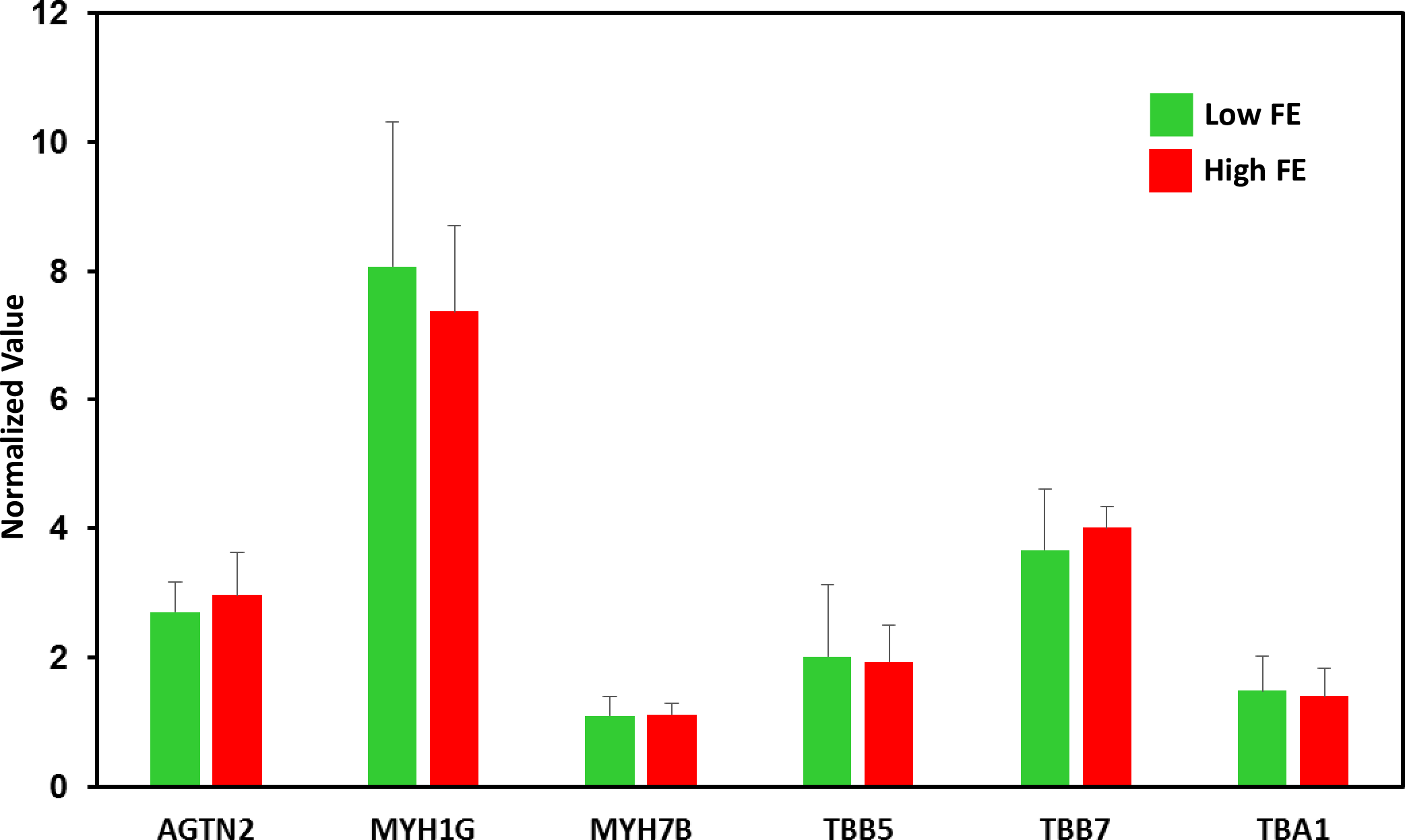

Supplement: S1 Fig — Proteins include: AGTN2 (alpha-actin 2), MYH1G (myosin heavy chain 1 gamma), MYH7B (myosin heavy chain 7 beta), TBB5 (tubulin beta 5 chain), TBB7 (tubulin beta 7 chain), TBA1 (tubulin alpha 1 chain). Values represent the mean ± SE (n = 4). (TIF) [file pone.0155679.s001.tif]

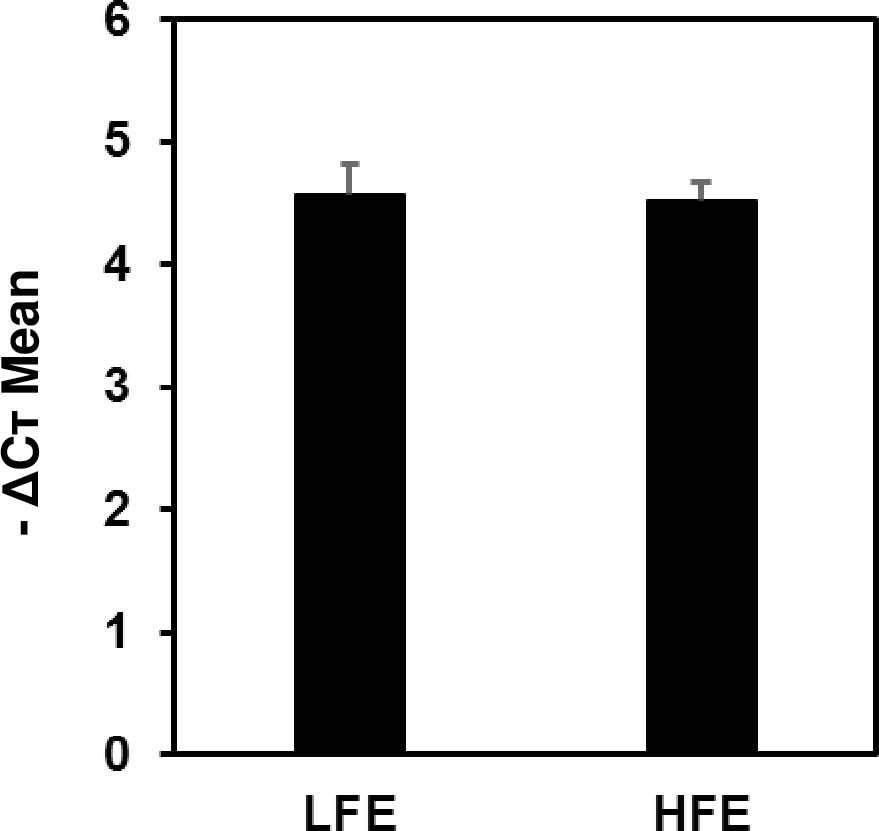

Supplement: S2 Fig — Values represent mean ± SE (n = 4). Primer information: mtDNA-D loop Forward—ACACCTGCGTTGCGTCCTA; Reverse—ACGCAAACCGTCTCATCGA; 18S rRNA gene Forward—TCCCCTCCCGTTACTTGGAT; Reverse—GCGCTCGTCGGCATGTA. (TIF) [file pone.0155679.s002.tif]
